# CT Scans: Balancing Health Risks and Medical Benefits

**DOI:** 10.1289/ehp.120-a118

**Published:** 2012-03-01

**Authors:** Charles W. Schmidt

**Affiliations:** **Charles W. Schmidt**, MS, an award-winning science writer from Portland, ME, has written for *Discover Magazine*, *Science*, and *Nature Medicine*.


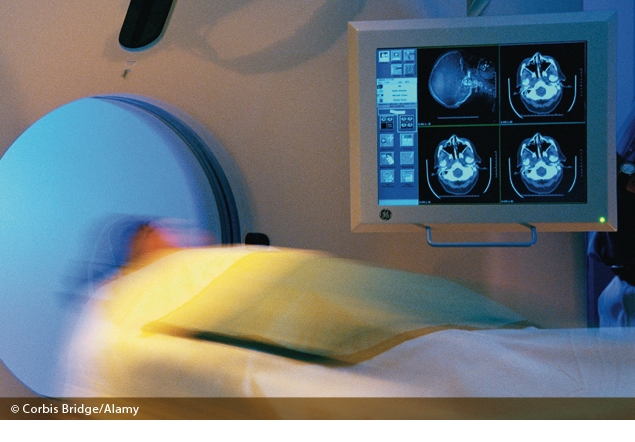
Computed tomography (CT) has been a boon for medical care. By generating detailed anatomical pictures, the technology can improve diagnoses, limit unneeded medical procedures, and enhance treatment. However, CT scans also dose patients with ionizing radiation, a known human carcinogen, posing a potential downside for public health. Mounting health worries over radiation risks are now driving efforts to limit avoidable CT scans and to reduce radiation doses where possible. “There’s a national focus on this issue right now,” says Marilyn Goske, a professor of radiology at Cincinnati Children’s Hospital Medical Center and chairwoman of the Image Gently campaign, a pediatric education and awareness campaign from the Alliance for Radiation Safety in Pediatric Imaging.

In December 2011 the Institute of Medicine (IOM) published a report concluding that ionizing radiation contributes more to the development of breast cancer than any other type of routine environmental exposure.^1^ About half the U.S. annual exposure to ionizing radiation comes from natural sources, including cosmic rays, but most of the rest comes from medical imaging and from CT scans in particular.^1^ The IOM cited research by Amy Berrington de González, a senior investigator in the Radiation Epidemiology Branch of the National Cancer Institute (NCI), whose calculations suggest that the CT scans performed in the United States in 2007 might produce up to 29,000 cancers in the future, about 6% of them in the breast and the remainder in the lungs, brain, and other organs.^2^

But the spotlight on CT safety has also drawn a backlash from those who say the risks are overblown. On 13 December 2011 the American Association of Physicists in Medicine (AAPM) issued a statement claiming that risks from CT imaging are “too low to be detectible and may be non-existent.”^3^ The AAPM added that “speculative predictions about cancer incidence and death” should be discouraged because they generate sensationalist media coverage that deters some patients who need CT scans from having them.

Donald Miller, acting chief of the Diagnostic Devices Branch of the U.S. Food and Drug Administration (FDA) Center for Devices and Radiological Health, cites 2 basic principles for decreasing CT radiation risks. One is justification, which refers to prescribing a CT exam only when it is medically necessary. The other is optimization, which refers to adjusting and operating a CT scanner so that images adequate for diagnosis are obtained at the lowest possible dose. Justification is more difficult to address, Miller says, because it involves case-by-case decisions made by individual clinicians. More attention has been paid to optimization, he says, but both principles are equally important.

## Calculating Risks

CT scanners were developed during the 1960s by England’s EMI Central Research Laboratories. Interestingly, EMI’s parent company owned a record label whose superstar act—the Beatles—generated funding that helped propel CT scanners into routine medical use.^4^ The technology started becoming widespread during the 1980s, and uses rose dramatically when faster scanning speeds made it possible to image large sections of anatomy in seconds.

An estimated 75 million CT exams were performed in the United States in 2009.^5^ Various estimates suggest that anywhere from 5% to 30% of these exams—each costing hundreds to thousands of dollars—may be medically unnecessary. Why would a clinician request an unnecessary scan? In a 2010 perspective article Rebecca Smith-Bindman, professor in residence at the University of California, San Francisco, School of Medicine, explained, “[T]here are few evidence-based guidelines regarding their appropriate use, and institutional use varies widely, reflecting physicians’ preferences and manufacturers’ promotion of these capabilities, rather than scientific evidence of improved clinical outcomes. . . . Ironically, technical improvements have led to increases in the identification of incidental (and almost certainly irrelevant) findings that result in follow-up CT for surveillance.”^5^

CT scanners emit X rays. Different tissue types absorb X rays in varying amounts, and the resulting contrasts provide detailed images of anatomy and disease. Absorbed radiation can break chemical bonds in tissues, liberating charged ions (hence the term “ionizing radiation”) that can damage DNA and produce cancer should cells be unable to repair themselves. Nonionizing radiation—lower-energy radiofrequency waves such as those emitted by microwave ovens and cell phones—doesn’t break chemical bonds.

Scientists can’t state conclusively that CT scans cause cancer until ongoing prospective studies of that link generate results. In the meantime, they estimate cancer outcomes using dose–response models derived from other radiation-exposed groups, such as atomic-bomb survivors and patients treated with radiation.

The dominant risk assessment model appears in a 2006 report from the National Research Council’s Biological Effects of Ionizing Radiation (BEIR) subcommittee.^6^ The BEIR VII model postulates there is no safe level of ionizing radiation exposure; carcinogenic effects are assumed to follow a linear dose response, meaning even the smallest exposure carries some level of cancer risk. The BEIR VII model generates so-called lifetime attributable risk (LAR) factors, which estimate the likelihood of cancer in hypothetical individuals as a function of dose. Multiplying the LAR by the number of people exposed to a given dose yields an estimate of expected cancers from that exposure in the population.

Berrington de González relied on BEIR VII to derive her estimate of 29,000 additional cancers resulting from CT scans performed in 2007.^2^ Likewise, Smith-Bindman used it to estimate that 1 cancer might appear for every 270 middle-aged women who undergo CT coronary angiography, a high-dose diagnostic procedure that scans heart vessels repeatedly after injection of a contrast dye.^7^ Young people face especially high risks, Smith-Bindman says, in part because they live long enough for cancer to develop after a carcinogenic exposure. Therefore, she estimates that women aged 20 who undergo the same coronary procedure have twice the risk as middle-aged women.^7^

On the flip side of the risk spectrum, Smith-Bindman also estimates that 1 cancer could appear for every 11,080 men who get a routine head CT scan.^7^ Head scans involve less risk in part because they dose a single organ—the brain—unlike coronary and abdominal scans that dose multiple organs, including the breasts and lungs.

## Debating Low-Dose Risks

Radiation’s low-dose effects remain controversial, however, and many experts question these high-end risk estimates. John Boice, a professor at Vanderbilt University School of Medicine, points to a paper published in 2011 reporting that human cells can efficiently repair radiation-induced double-strand DNA breaks.^8^ The paper’s authors claim their findings cast doubt on the assumption that risks from ionizing radiation are proportional to dose. “And that implies that radiation could have a no-effects threshold,” Boise says. “At the very least it suggests that low-dose effects are lesser than those predicted from high-dose extrapolation based on a linear model.”

Likewise, William Hendee, a professor at the Medical College of Wisconsin, who authored the AAPM’s position statement on CT risks, argues that cancer estimates from CT exposure typically draw on worst-case assumptions at every step of the process, from expectations of dose linearity to the numbers selected for calculating LARs. “Every parameter in the BEIR VII model has a distribution of possible values,” he says. “So the actual number of predicted cancers could range from the highest possible number to zero.”

Hendee also claims that BEIR VII relies excessively on data generated by atom-bomb survivors, many of whom suffered from malnutrition, lack of medical care, and other effects resulting from the blast, he says. “No one knows the cumulative effect of those risks compared with the radiation risk,” Hendee says. “And cancer incidence differs for Japanese and U.S. cohorts—more stomach cancer in the Japanese, and more breast cancer among Americans, for example—so the two populations aren’t really comparable.” Finally, atom-bomb survivors received instantaneous full-body radiation doses of varying magnitude (depending on distance from the blast and other factors), Hendee explains, unlike CT scan recipients, who get much smaller, targeted exposures from imaging.

The AAPM’s position statement asserts that cancer risks are negligible at effective doses below 50 millisieverts (mSv) for single CT exposures and below 100 mSv for multiple exposures over short durations. But a 2003 paper coauthored by David Brenner, director of the Center for Radiological Research at Columbia University School of Public Health, claims that the atom-bomb survivor data work well for low-dose extrapolation because they’re drawn from large cohorts with well-defined exposures and complete followup.^9^ And those data, Brenner says, show a statistically significant trend of increasing cancer risk with increasing organ dose between 5 and 100 mSv. Smith-Bindman echoes his conclusions, arguing that cancer risks are established even at effective doses of 10 mSv.

Estimates of the average effective dose from CT scans vary widely. Smith-Bindman’s calculations, based on data supplied by 6 integrated health plans that kept detailed dose information for individual patients, indicate that the average effective dose of ionizing radiation from medical imaging per year in the United States was 6.7 mSv in 2007, most of it from CT.^1^ Meanwhile, by dividing the total amount of radiation emitted by CT scans in 2006 by the total U.S. population, the National Council on Radiation Protection & Measurements calculated a much lower average per-capita effective dose of 3.0 mSv. Miller argues that because it’s based on a much broader data set, the council’s estimate is more accurate.

## A Data Vacuum

Of course, a single CT scan can deliver doses that far exceed these annual averages. For instance, Smith-Bindman reported that a CT angiogram can deliver an organ-specific dose to the breast of 51 milligrays (mGy)^10^ or greater.^7^ But doses per indication vary by institution and by the patient’s size, and national averages simply aren’t available. “If you were to ask, ‘What’s the average radiation dose for a head scan in the U.S.?’ the answer would be, ‘We don’t know,’” says Michael McNitt-Gray, an associate professor of radiological sciences at the University of California, Los Angeles.

Part of the problem is that radiologists have yet to reach a consensus for how to define the dose to the patient, he says. Effective and organ-specific doses both relate to how much radiation the body absorbs. But CT scanners don’t report the absorbed fraction. Instead, they report only what the machine emits, which is less than what’s absorbed by the body. One commonly reported value, the computed tomography dose index (CTDI), describes the amount of radiation given off by the CT scanner during a single scan, or “slice.” Another metric—the total dose-length product (DLP)—describes the amount of radiation given off for all the slices in a CT sequence during an examination.

These units provide a useful way to compare scanner output, but since they don’t describe dose, they’re not adequate for risk assessment, Miller says. “The real goal is to calculate organ doses,” he says. “If we know the organ dose, we can do a much better job estimating the individual’s cancer risk.”

The American College of Radiology (ACR) and other medical professional organizations have recommended “reference levels” for some medical imaging exams. Reported as CTDI, these levels represent upper-bound radiation exposures for specific medical indications that shouldn’t be exceeded among individual patients. But the reference values aren’t enforced by the federal government, whose regulatory authority extends only to CT scanner manufacturers, not to imaging protocols designed by facilities that use the technology. “So you could get a CT scan at one hospital, and the radiation dose could be ten to a hundred times higher than it would be somewhere else,” Smith-Bindman says.

In 2009, 206 patients at Cedars-Sinai Medical Center in Los Angeles accidentally received brain-specific overdoses ranging up to 6,000 mGy while being scanned for suspected stroke.^11^ Facing litigation, the hospital released only limited information about what led to the overdoses, claiming they occurred when technicians bypassed the protocol that came installed on the machine. In response, California legislators passed SB 1237,^12^ which represents the first attempt to require reporting of radiation doses from CT in the United States. The law stipulates that as of 1 July 2012, California hospitals must record in an electronic database the dose (as either CTDI or DLP) of every CT scan given. In addition, the scanner output must be reviewed once a year by a medical physicist.

Other overdosing episodes have since been disclosed elsewhere in the United States, and additional states are now considering legislative or regulatory changes inspired by the California model, according to Ruth McBurney, executive director of the Conference of Radiation Control Directors, based in Frankfurt, Kentucky. McBurney said her organization will be surveying state initiatives within the next few weeks.

## Addressing Problem Areas

The FDA’s main efforts in the area of radiation protection revolve around its Initiative to Reduce Unnecessary Radiation Exposure from Medical Imaging, which launched in 2010.^13^ Miller says the FDA is making progress on the initiative by working with industry on new equipment safety features, working with AAPM and others to establish “reasonable scan protocols and standardize imaging terminology,” devising safety checklists for CT operators, and funding efforts to improve training for equipment operators involved in pediatric CT imaging, among other activities.

Meanwhile, the ACR has set out to tackle the dose variability problem by creating a national registry that allows facilities to upload anonymized scan information for different procedures into a centralized database. Facilities can check their dose values against those of other participating hospitals as a quality control tool to evaluate their own imaging practices and dosing protocols. Over time, the range of doses delivered for a given exam should narrow toward an average value, according to Richard Morin, the registry’s chairman and a professor at the Mayo Clinic campus in Jacksonville, Florida.

If there’s a problem with the ACR registry, it’s that it only addresses CT optimization, or efforts to reduce CT examination doses. In its current form it cannot address the broader issue of justification, or how clinicians decide if a CT scan is medically necessary, Miller says. So in December 2011 the FDA partnered with the Centers for Disease Control and Prevention to sponsor a symposium aimed at investigating prospects for a national registry that might tackle both these angles.^14^ Conclusions from that meeting, which was hosted by the National Academies as part of its annual Beebe Symposium, will be contained in a forthcoming report, Miller says.

Later in 2012 investigators will release results from the first study of CT-related cancer in children. Led by the NCI and the University of Newcastle, the study follows 200,000 U.K. residents who received CT scans before the age of 18 between 1985 and 2008. “We’ve been following them for brain cancer and leukemia, which are typical childhood cancers,” says Berrington de González, the study’s principal investigator at the NCI.

According to Berrington de González, doctors in the United Kingdom prescribe about 7 times fewer CT scans than those in the United States, French doctors prescribe 3 times fewer, and in Germany, half as many scans are given. Asked why, she says, “That’s the million-dollar question! It’s not entirely clear, but economic incentives and defensive medicine may be some of the factors that account for the particularly high uses here.”

Still, Boice points out that CT risks must always consider and be balanced against the substantial clinical benefits—CT scans can save lives by improving diagnoses and even reduce some medical costs if they limit unnecessary procedures, such as exploratory surgery. What’s more, CT technology has been linked to declines in cancer mortality, according to the ACR. In children, decreases in exploratory surgeries and decreased time to triage patients are direct benefits of CT scans. “They’re only a problem if the examinations are unnecessary or if the exposures are higher than necessary to provide a good clinical image,” Boice says.

“When clinically justified, a CT scan’s benefits always outweigh its associated individual risks—whatever they are,” says Brenner. “That being said,” he adds, “even when a CT is justified we should still use the lowest reasonably achievable dose for the scan.”
